# An Experimental Study of Mortars with Recycled Ceramic Aggregates: Deduction and Prediction of the Stress-Strain

**DOI:** 10.3390/ma9121029

**Published:** 2016-12-21

**Authors:** Francisca Guadalupe Cabrera-Covarrubias, José Manuel Gómez-Soberón, Jorge Luis Almaral-Sánchez, Susana Paola Arredondo-Rea, María Consolación Gómez-Soberón, Ramón Corral-Higuera

**Affiliations:** 1 Barcelona School of Civil Engineering, Polytechnic University of Catalonia, C. Jordi Girona 1-3, Building C2, 08034 Barcelona, Spain; guadalupe.cabrera04@gmail.com; 2 Barcelona School of Building Construction, Polytechnic University of Catalonia, Av. Doctor Marañón 44-50, 08028 Barcelona, Spain; 3 Faculty of Engineering Mochis, Autonomous University of Sinaloa, Fuente de Poseidón y Ángel Flores s/n, Col. Jiquilpan, Module B2, Los Mochis, 81210 Sinaloa, Mexico; jalmaral@uas.edu.mx (J.L.A.-S.); paola.arredondo@uas.edu.mx (S.P.A.-R.); ramon.corral@uas.edu.mx (R.C.-H.); 4 School of Civil Engineering, Metropolitan Autonomous University, Av. San Pablo No. 180, Col. Reynosa Tamaulipas, Delegación Azcapotzalco, 02200 Distrito Federal, Mexico; cgomez@correo.azc.uam.mx

**Keywords:** mortar, ceramic wastes, recycled aggregates, stress-strain behavior, numerical simulation

## Abstract

The difficult current environmental situation, caused by construction industry residues containing ceramic materials, could be improved by using these materials as recycled aggregates in mortars, with their processing causing a reduction in their use in landfill, contributing to recycling and also minimizing the consumption of virgin materials. Although some research is currently being carried out into recycled mortars, little is known about their stress-strain (σ-ε); therefore, this work will provide the experimental results obtained from recycled mortars with recycled ceramic aggregates (with contents of 0%, 10%, 20%, 30%, 50% and 100%), such as the density and compression strength, as well as the σ-ε curves representative of their behavior. The values obtained from the analytical process of the results in order to finally obtain, through numerical analysis, the equations to predict their behavior (related to their recycled content) are those of: σ (elastic ranges and failure maximum), ε (elastic ranges and failure maximum), and Resilience and Toughness. At the end of the investigation, it is established that mortars with recycled ceramic aggregate contents of up to 20% could be assimilated just like mortars with the usual aggregates, and the obtained prediction equations could be used in cases of similar applications.

## 1. Introduction

The recycling of waste products from the ceramic industry is an important objective, as they are an obstacle to achieving a sustainable society [1]. This industry produces bricks, paving and roof tiles, of which 2% are taken to landfill due to deficiencies, cracks or fissures. This makes their controlled elimination difficult and as a result they end up being the origin of a serious environmental problem [2,3].

As a consequence of the search for solutions to their processing and recycling, recycled ceramic aggregates (CA) are being researched as total or partial substitutes for the usual aggregates (UA) (in mortars as well as in concrete); these new applications use fractions of both coarse and fine sizes [4], and also as a partial substitution of cement itself (sporadically reaching the adequate properties [5,6,7,8,9,10,11,12,13,14]). In short, all of this research contributes to recycling, to a closed life cycle in building materials, reducing the environmental impact by preventing the waste products from reaching the landfill, and minimizing the consumption of natural resources [15,16].

CA should be understood as materials that proceed from a previous use (rejects from a ceramics factory, or from the demolition of architectural elements containing ceramics) and which ultimately may be considered as residues; if they are subjected to processing (selection, crushing and sieving) they are transformed, allowing their inclusion in other uses. In comparison with the CA, the UA are materials without a previous use (extracted from quarries or riverbeds), typically used in the construction industry to make concrete and mortars (sands). Therefore, when CA is used to make a new mortar (replacing the UA), the resulting Mortar generated contains recycled aggregates, in this particular case of ceramic origin (RCM).

Recent research into mortars with different replacement percentages of UA by CA aggregates has concentrated its results on explaining their behavior in their fresh and hardened state, thereby trying to establish their real chances of possible practical application.

Regarding their fresh state, Bektas et al. [17] reports that the consistency of the RCM is modified according to the CA content in the coarse fraction. This behavior was explained by the high water absorption capacity of the CA (also validated by [18]). If only the fine fraction of the CA is used, Silva et al. [19] reported the opposite, concluding on this occasion that the mixing water needed for the determined consistency decreases as the fine CA are increased; it is the size and content of the CA used which dictates this property on this occasion (this is also verified by previous results [20]).

Ay and Ünal [21] conclude in their study that the use of the CA in its fraction of ceramic powder (CePo) (as a cement replacement) increases the consistency. On the contrary, Turanli et al. [10], needed to increase the amount of water; however, in both cases the tolerance is acceptable according to ASTM C618. In a similar manner, Pereira-de-Oliveira et al. [14] obtained a decrease in the consistency of both brick and roof tile CA, attributing this to a theoretical increase in the specific surface area of the mortar (fineness of the CePo).

Therefore, if the use of CAs in the RCM leads to a loss in their consistency, it can be expected that their modification (water content available for forming hydration products) may affect their later behavior in their hardened state. Thus, with an adequate consistency, a foreseeable improvement in resistance can be guaranteed (compacting, which produces a higher density); the common thread of the behavior being that good consistency leads to high density, which in turn guarantees good resistance (desirable elastic behavior). Referring to the density of the RCM, factors such as the size of the aggregates [19], the amount of replacement [16,18,22] and its contribution as a cementing material have been studied [16].

With regard to the properties of the RCM in their hardened state, the apparent density has experienced a similar trend to the density in fresh state, showing decreases in density as the CA replaces the UA [16,18,22]. This is explained by the lower density of the CA; therefore, it is to be expected that, in the σ-ε behavior of the RCM, the replacement factor (density reduction) will also depend on this.

Porosity in the RCM has also been previously assessed [9,12], the RCM with finer CA show greater porosity [4]; referring to the control mortar and analyzing the distribution of its pore sizes, it is shown that the micropore zone is reduced and in the mesopore zone the values are similar in both mortars, eventually increasing in the macropore zone, generating the most notable difference in the mortar with CA. Therefore, it should be emphasized that the structure or porous network of a mortar should, intuitively, be a direct factor by which the transfer of tensions must be established; this being, therefore, a property which influences their elastic behavior and causes limitations in their resistance (possible deformation and low resistance capacity), or on the contrary favors it (increase in matrix rigidity).

Some properties have been established regarding the mechanical behavior of the RCM, among which three principles for compressive strength (fm) have been established: replacement of UA by CA, of UA by CA and use of CePo for a part of the cement, and of CePo by cement.

In investigating the total substitution of UA by CA, different studies have been carried out [4,23], in which losses of up to 39% (justified by the lower specific weight and higher absorption of the CA) and from 40% to 73% (caused by different particle sizes-crushing method) have been obtained at an age of 28 days. Contrary to previous research [24], increases in resistance (14%) have also been established, without being conclusive.

Research has also been carried out into the partial replacement of UA by CA [16,18,22], concluding that for percentages below 30% (in some cases even 50%) the fm is equivalent or even superior to the reference sample. This increase is due to the pozzolanic reaction that the fine fraction of the CA may show, as well as other physical effects (absorption, form and roughness). Above this “limit” of CA content, a resistance loss of up to 14% should be expected; this may be the result of the dosage method used (by volume), which underestimates the high porosity of CA.

Regarding the second principle of the research, it is concluded that RCM in which 100% of the UA is substituted by CA, as well as 10%, 20% and 30% substitutions of cement by brick CePo, cause increases in resistance of between 11% and 3% (10% and 20% in brick CePo) [24]; on the contrary, there is a reduction of 37% if the 30% mix is used. These results are explained by the possible pozzolanic reaction of the CePo; however, not all the implications of this work have been clarified with precision.

Finally, for the third principle, up to 40% replacement of brick CePo for cement has been used, revealing decreases in compressive strength when the percentage of CA substitution increases [16,21]. Contrary results have also been reported, showing increases of 3% (water/cement (*w*/*c*) = 0.4) when 20% of the cement was replaced [12]. On these occasions, it seems that the RCM benefitted from the *w*/*c* effect, for in an identical test but using *w*/*c* = 0.5 and for whatever percentage of replacement, the results always showed a loss of resistance. In a final study, results were obtained which differed from all the previous ones; for a replacement of 30% of CePo, reductions of up to 42% were reported [9].

Several hypotheses can be obtained from all the previous studies, although none are conclusive. It seems that variables such as the cooking temperature of the clay CA (potential pozzolanic capacity), the speed of its reaction or maturity (hydration process) and the blocking effect in the cementing matrix of the RCM (high density and closure of the porous network) may be involved in this behavior, but given the divergence and variability of the results and variables that intervene, further research is necessary. On the other hand, it may be said that whatever the mechanism—chemical, physical or mechanical—that alters the properties of the compressive strength of the RCM, this will have a direct repercussion on their deformation behavior, as this connection in their behavior has already been established in the general ambit of concretes and mortars [25,26,27], and should also arise in the RCM.

Flexural strength is another property of interest in the study of the σ-ε behavior in agglomerate materials, which in the case of the RCM is expected to show particular considerations. Favorable replacements of CA for UA with values of up to 50% have been reported, without affecting the flexural strength [18,22]. In the case of 100% substitution, losses of 6% were reported [4,23]. The previously mentioned strength increases are explained either by the contribution of the pozzolanic effect of the CA, or by physical effects such as increases in porosity and angularity, which cause the “sticking effect” of the cement paste; this contributes to the increase in strength. However, with the use of 100%, this behavior can be understood due to the fact that in the case of high CA contents the beneficial pozzolanic effect does not predominate in the behavior of the cementing matrix; it is the low specific weight, and high absorption and porosity, which finally establish this as “the weak link” of the group.

In the event of using CA to replace cement [9,21], strength losses have been reported for all the replacement percentages. In this case, it is clear that the substitution of CA for cement is not equal to the hydration capacity of the latter; however, the application may be justified for use with low contents (assumable losses of strength) due to the value of its environmental benefits.

Regarding the static σ-ε relationship of the RCM, little has been explained with respect to their habitual characteristics, such as the establishment of either the plastic or the elastic range (Young’s modulus, or the modulus of Elasticity (*E*)); the same applies regarding their respective elastic deformation (ε_elastic_) established at 40% of the maximum load of failure (0.40 fm), and their maximum deformation of failure (ε_max._) reached by the material before the failure is present (fm). Despite this, these parameters are considered fundamental in evaluating the mechanical characteristics of the materials [28], and necessary for the design of the constructive elements in which they are integrated; therefore, an eventual proposal of standardized use with mechanical objectives requires their study, determination and explanation.

**Figure 1:**
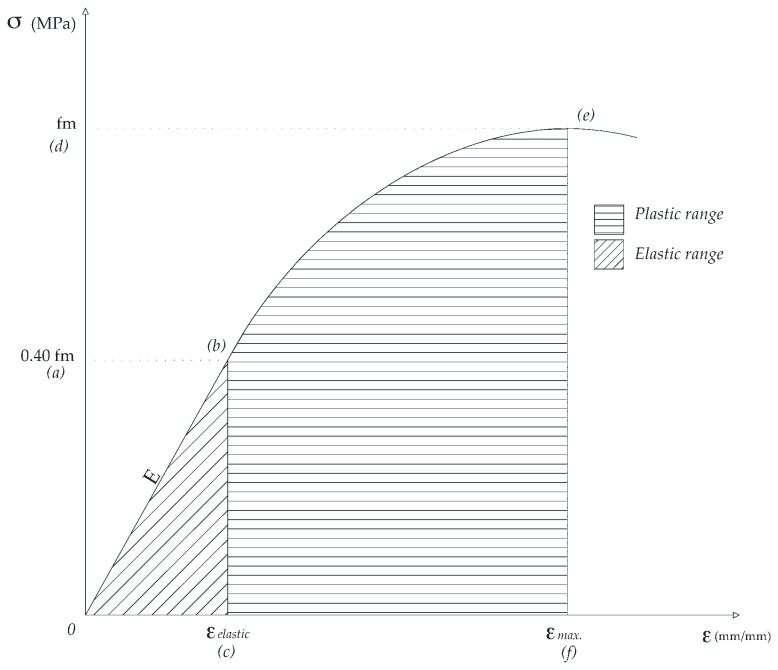
σ-ε curve diagram.

By general agreement, *E* has been nominated as the ratio between the increase of strength and the corresponding change to the unitary deformation of a material [29]; if the level of strength exceeds the elastic limit, then the material enters the plastic range, characterized by displaying non-proportional increases in its deformation as a response to the increased stress to which it is subjected. When the applied charge produces the failure in the material, the deformation at this instant is specified as the ε_max._ of the failure (see Figure 1).

Static tests are the habitually used procedures for determining the σ-ε relationship of the materials, this being a mechanical characteristic, which leads to defining them in several aspects (elasticity, plasticity, malleability and hardness). The diagram σ-ε is unique for each particular material; however, these can be classified into two different types of materials: ductile materials (showing large ε) and fragile materials (showing small ε) [30]. From the diagram σ-ε it is possible to determine the *E* (“desirable” behavior of construction materials), which describes its relative rigidity. This property can be obtained from a laboratory test in which the slope of the elastic region of the diagram σ-ε [31] is determined; numerically this is obtained from the coefficient of the strength regarding the unitary elongation [32] as can be seen in Equation (1):

(1) $$E=\sigma /\varepsilon =\frac{F/A_{0}}{\Delta L/L_{0}}=FL_{0}/A_{0}\Delta L$$

where *E* (in MPa) is Young’s Modulus; *F* (in N) is the applied axial force; *A*_0_ (in mm^2^) is the area of the original transversal section; Δ*L* (in mm) is the variation of the longitudinal dimension of the object; and *L*_0_ (in mm) is the original height.

Regarding previous results of *E* for the RCM, some particular variables of their study have been reported. The first was the partial use of CA as a substitution for cement in percentages with maximum values of up to 40%. Moriconi et al. [9], applying static compression tests and with the diagram curve σ-ε, showed losses at all ages of the study, the maximum being at 28 days (46% with respect to the reference); with similar results. Toledo Filho et al. [12] reported that *E* decreases inversely to the increase in the CA replacement percentage; with substitutions of 0%, 10%, 20%, 30% and 40%, decreases of 3%, 5%, 7% and 9%, respectively, regarding the control mortar (at 28 days, *w*/*c* = 0.5) were observed. However, with *w*/*c* = 0.40, similar behavior to the previous was obtained, with the exceptions being mortars with 10% and 20% CA, which showed an increase in *E* of 1% and 2% respectively, both with reference to the control mortar. Therefore, it seems that the replacement factor is adverse and the *w*/*c* relationship is less decisive (within the studied limits).

The second variable studied was the use of RCM with CA replacing different percentages of UA, and in which losses have also been reported; for example Silva et al. [20] reported decreases of *E* when 10% of CA was replaced (the variables of cement/aggregate (*c*/*a*) studied were = 1:6 and 1:4 ), indicating as the most important result that the effect of *c*/*a* could be similar (18% and 17% of losses respectively) at prolonged ages (2–5 months); this behavior was held responsible for the formation of fissures, showing that a lesser *E* permitted lower internal tensions for identical deformations. In parallel works [18], an experimental campaign was defined with *c*/*a* = 1:4 but now attaining replacement values of CA by UA of 50%; on this occasion the losses indicate values of up to 40% with respect to the reference sample (at both two and five months), concluding that—if these losses are accepted—the application of monolayer mortar to the walls may be appropriate.

In the third variable studied, total replacements of CA were made, both for UA and for the cement; Higashiyama et al. [24] used two different particle size (PS) of CA in their study, using 100% CA as an aggregate and studying replacements of 10%, 20% and 30% for cement. On this occasion, for the RCM with 10% and 20% cement replacement, increases of 2% and 5%, respectively, were obtained with regard to the reference samples, this behavior being explained by the good mechanical qualities of the CA; however, in the case of the variable that used 30%, losses of 6% were reported, for reasons that have not been fully clarified.

Based on the previous studies, the objective of this research is to determine more precisely all the σ-ε relationships that the RCM could contain due to the effect of the replacement of their UA by different percentages of CA. Consequently, the experimental methodologies have been established for: aggregate substitution procedures, mortar mixing protocol, static mechanical and deformation spotting tests and, finally, the numeric analysis of data, which allows the real values of their σ-ε behavior to be established, such as the fm, 0.40 fm, ε_elastic_, ε_max._, *E*, toughness (*T*) and resiliency (*U*_r_), as well as their particular prediction equations.

## 2. Materials and Methods

### 2.1. Materials and Mortar Dosage

**Figure 2:**
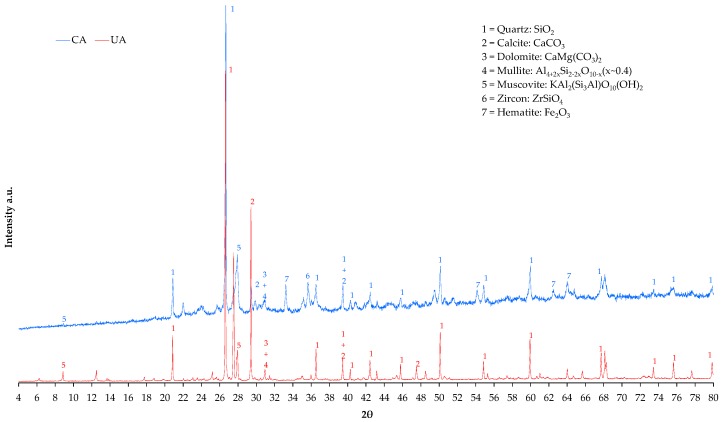
XRD diffractogram for the CA and UA studied.

CA was available as an aggregate for use in the study of the RCM, obtained from a treatment plant for aggregates from waste and demolition, which in turn came from a local company that supplied ceramic roof tiles for the construction industry; the material was eliminated from the production process for not meeting the requirements (defects in size or faults in geometry). The material used had a PS of 0–5 mm. A silica sand, from a local supplier of natural aggregates for construction, was used as UA (PS of 0–4 mm). Figure 2 shows the results of the X-ray diffraction tests (XRD) (brand PANalytical X’Pert PRO MPD Alpha1 diffractometer, Almelo, The Netherlands) equipped with a source Cu Kα_1_ radiation (λ= 1.5406 Å) X-rays; the test was performed in the range 4° to 80° of 2θ in steps of scanning 2/s. For both aggregates used in this research, the characteristic peaks of the principal components identified are marked on the graph, demonstrating that both aggregates can be considered “equivalent” regarding the presence of mineralogical compounds. They only differ regarding the peaks of Zircon and Hematite present in the CA (both common in ceramic materials due to their refractory capacity and pigment), but without them being a cause of distinction in later behavior due to their alternative use.

The physical properties of the aggregates used are shown in Table 1; the bulk density values in oven-dry condition (*M*_OD_), the bulk density in saturated-surface-dry condition (*M*_SSD_) and the void content correspond to the materials used; on the other hand, the density values in oven-dry condition (*D*_OD_), Density in saturated-surface-dry condition (*D*_SSD_), absorption (more significant in this case), fineness modulus and particles of <75 µm correspond to the aggregates finally used in the research—the granulometric profiles adjusted to the limits of ASTM C144 [33]—which involves dividing the original profiles into two fractions (sieve No. 30, 0.59 mm) in the search for the maximum bulk density. This permits two different granulometric profiles to be adjusted, thereby ensuring they are more equivalent. The CA was made up of 60% of the material retained in the sieve and 40% of the material that passed through; in the case of the UA the optimal adjustment was made using 50% of each material, the retained and the sieved.

Of the tests carried out, in agreement with others [16,22], the density of the CA was inferior to that of the UA, in general being an average 24% less. The opposite was the case for absorption, in which the CA were up to 12 times bigger than the UA, similar to others reported previously [4,18,22].

Portland cement CEM I 42.5 N/SR (UNE EN 197-1:2011 [37]) was used as a binder, being commonly used and having the usual properties and components. Potable tap water was also used in making the mixtures.

### 2.2. Experimental Campaign/Specimens

Test specimens of 4 cm × 4 cm × 16 cm were made with different material contents, and the percentages of CA used to substitute the UA were identified as: usual mortar (UM), which refers to the mortar in which only UA was included (0% CA), and RCM XX%, which refers to the RCM in which a part of the UA is replaced (XX%, in weight) by CA. Thus, XX% represents the percentage of CA (made up of the two fractions of particle sizes, 60% > sieve No. 30 and 40% < sieve No. 30), and its complement, until the total amount of material used represents the percentage of UA (made up of the two fractions of particle sizes, 50% > sieve No. 30 and 50% < sieve No. 30). The total of the study variables to be compared with the UM were designated thus: RCM10, RCM20, RCM30, RCM50 and RCM100 (10%, 20%, 30%, 50% and 100%).

Table 2 presents the characteristics and proportions for obtaining 1 dm^3^ of each of the study mixes used in the research; all the mixtures were designed with an initial *c*/*a* ratio of 1:4 and of *w*/*c* = 0.5. The dosage process was carried out in accordance with criteria generally accepted in previous studies: the recycled aggregates required previous saturation to prevent the movement of water necessary for hydration [38,39].

The absorption of the aggregates used (in the case of ceramics, and depending on the XX%) will cause that the final *w*/*c* relation varies. However, this increase in *w*/*c* should not be interpreted as a direct increase in the water used chemically to react with the cement (forming crystals or composites), but as mixing water which will be used to saturate the recycled aggregates (with more absorption capacity due to their open porous structure) and prime them for mixing (humid, superficially dried) with an identical flow capacity (ASTM C109/C109M-05). Although it would seem initially that these mortars are not suitable for comparison, they are if the implications of substituting one group of aggregates for another are taken into account. Equally, their specific and particular characteristics play a part in designing the mixture, as well as their equivalences in the behavior.

The mixing sequence and the duration of the process were in accordance with the ASTM C305-06 norm [40], preparing the initial sequence for adapting the particular saturation requirements of the CA: introducing the UA and/or CA and the total water content of the mixture into the mixer (brand Matest, Mod. E93, Brembate Sopra, Italy), leaving the materials to soak for one minute before introducing the cement. From this point onward (8.1.3 of the ASTM C305-06), the rest of the process is standard. The compliance of the properties in fresh state (ASTM C230/C230M-03 [41], C231-08b [42]) is verified before molding the test specimens.

Once the mixing is completed the resulting product is introduced into the molds for curing, in accordance with ASTM C109/C109M-99 [43].

### 2.3. Details of Tests and Procedures

For determining the density of the RCM, the prescriptions of UNE EN 1015-10 [44] and UNE EN 1936 [45] were used, as well as the remaining half of a specimen previously subjected to a flexural test at an age of 60 days (considering that this property has reached stability of maturity at this stage).

The fm and the σ-ε curve were obtained at the age of 90 days (sufficient maturity for the aims of the study) in test specimens that had previously been removed from their curing process in water. The compression strength test was carried out in line with ASTM C349-97 [46].

**Figure 3:**
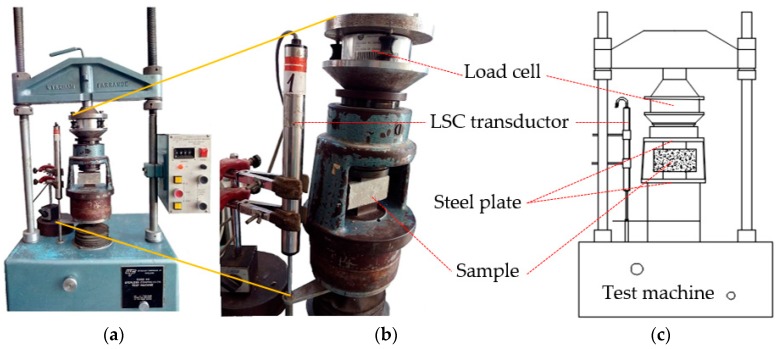
(**a**) General configuration of the experimental arrangement; (**b**) detail of LSC; and (**c**) idealized scheme from set.

**Figure 4:**
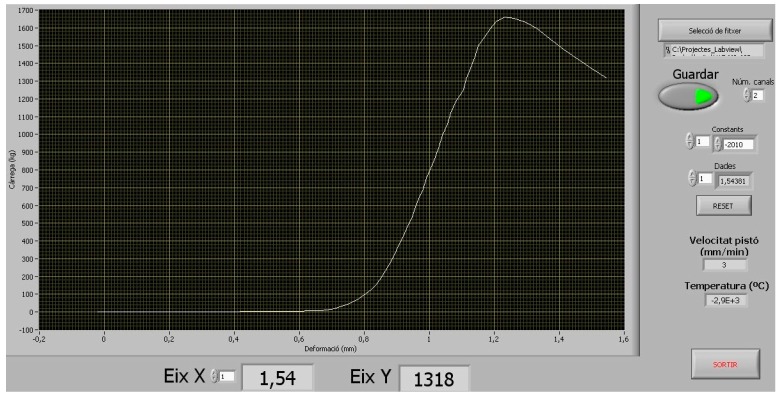
Curve σ-ε resulting from a test.

The σ-ε curve necessary for determining *E* was obtained during the compression strength test; for which it was decided to use a linear displacement sensor (Linear Strain Conversion (LSC) Transducer, APEK Desing & Developments LTD, MPE Transducers Division, Wimborne, Dorset, UK), connected to the universal press (5000 KG Stepless Compression Test Machine, Wykeham Farrance Ltd., Tring, UK) with the aim of establishing the deformation produced by the effect of the load on the specimen. An external load cell (5 t ± 2 mV/V, brand Hottinger Baldwin Messtechnik (HBM), Darmstadt, Germany) was used to establish the amount of charge applied to the specimen; both instruments (transductor and charge cell) were connected to a data acquisition unit (model SCXI-1000, brand NATIONAL INSTRUMENTS, Austin, TX, USA), which sends the acquired data to a computer; this is later processed and finally the graph of the σ-ε curve is generated. Figure 3 shows the experimental setup used, and in Figure 4 there is an example of the resulting graph, the vertical axis (*Y*) indicating the values of the charge in kg, and the horizontal axis (*X*) showing the deformation in cm.

Once the data had been extracted from the computer, they were processed on a spreadsheet to obtain the σ-ε curves in accordance with Figure 1. As a working principle of the data capture system itself, a manual adjustment was necessary, which involved locating the starting point of the test. This was established in the following manner: the first pair of values σ and ε in which an ε > 0 (σ_2_ with ε_2_ > 0) was detected was considered to be the second pair of values of the test, which therefore makes the previous values (σ_1_ and ε_1_) become σ_1_ = 0 and ε_1_ = 0. The rest of the pairs of σ and ε were then recalculated, in accordance with this principle of correction. The previous adjustment eliminates the initial semi-straight section of the curves, which had originated in the adjustments made at the start of the charge phase (adjustment of the charge plates, parallelism of the specimen faces, acquisition of LSC, etc.). On the other hand, it allows all the curves to be put at the same test starting point, thus making their comparison easier.

To continue with the information analysis, the value of fm was located, which was determined as the value of maximum tension reached prior to the tension decrease (specimen failure). Then the value of 0.40 fm was determined, and was established as the limit between the elastic and the plastic behavior of the material. In both cases the projection that arose between these values on the vertical axis (fm and 0.40 fm) and the intersection with the σ-ε curve allowed the values of ε_max._ and ε_elastic_, respectively, to be established on the horizontal axis.

For the simplified determination of *E* the following procedure was established: the slope of the theoretical straight line is obtained, created in the section limited by the intersection of the curve with 0.40 fm (point b of Figure 1) and the origin of the diagram (point 0 of Figure 1) [31]. Bearing in mind that the material deforms in the elastic range (without σ, its ε is recovered), the equation used to determine the slope was of linear type, with a constant slope. For its regression, all the pairs of values σ-ε contained within the previously established range were used. Equation (2) is used:

(2) y=ax+b

where *y* is the dependent variable (σ, in MPa), *a* is the slope of the curve (*E*, in MPa), *x* is the independent variable (ε, in mm/mm) and *b* is the point where the slope cuts the “*Y*” axis (in MPa).

The area below the σ-ε curve has been defined as *U*_r_ [47], assigning to this area the concept of the energy which the test specimen of mortar is able to absorb or transform (in deformation), with a validity limit of up to an σ of 0.40 fm. For a numerical quantification, the area below the curves, limited by the points “0”, “b” and “c” of Figure 1, was estimated.

Regarding *T*, this has been associated as the total energy absorbed or transformed in deformation by the test specimen of mortar of the entire curve σ-ε [31,48]; however, given the difficulties and implications of a correct capture of the section of the σ-ε curve after fm, only the area below the σ-ε curve, bounded by the points “0”, “b”, “e”, “f” and “c” in Figure 1, have been considered. In both cases the area below the curves is obtained by means of the trapeze method, according to Equation (3), for each pair of values of increase of σ and its respective ε, in order to get their sum and thereby obtain the total sought area below the curve.

(3) $$\overset{i}{\underset{0}{\int }}f\left(x\right)dx=\left(\varepsilon_{2}-\varepsilon_{1}\right)\left[\frac{\sigma_{1}+\sigma_{2}}{2}\right]$$

where ε_1_ (mm/mm) is the lesser deformation of the pair of study values; ε_2_ (mm/mm) is the greater deformation of the pair of study values; σ_1_ (MPa) is the lesser stress of the pair of study values; σ_2_ (MPa) is the greater stress of the pair of study values; and *f*(*x*) (MPa) is the area below the σ-ε curve. Similarly, the secant modulus of elasticity (*E*_0_) was determined from the origin of the coordinates to the corresponding point of maximum stress of its theoretical straight line. This was established by obtaining the slope of the theoretical straight line which is created in the section limited by the intersection of the curve with fm (point d) and the origin of the diagram (point 0) of Figure 1.

## 3. Results

Here now follow the results obtained regarding density in hardened state, the compression strength at *E*, *U*_r_, and *T*, as well as the values of ε_max._ and ε_elastic_ obtained from the experimental campaign described; a numerical-analytical process was then carried out with these results to determine the equations for predicting the behavior of the RCM for each of the studied properties, and finally the equations which define the theoretical behavior of the σ-ε curves were established.

### 3.1. Density in Hardened State

Table 3 shows the results of the density (ρ) obtained for the RCM studied; it can be noted how the RCM are always less heavy than the UM and, equally, their density is inversely correlative to the CA content. This is due to the density of the aggregates used (see Table 1).

### 3.2. Compression Strength

**Figure 5:**
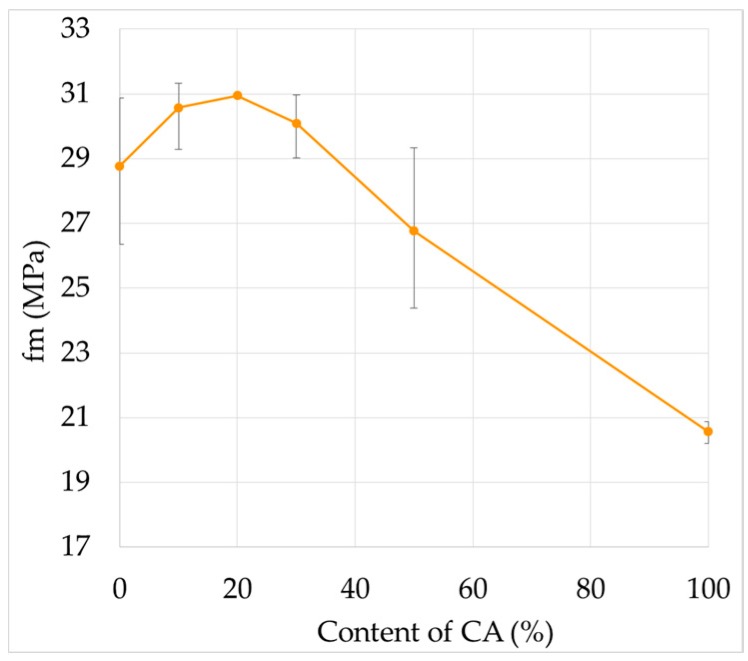
Compressive strength of the RCM at 90 days of age.

In Figure 5, the values obtained from fm are presented for the different RCM studied, in which three zones of behavior can be observed. First (low content), similar values to those of UM are obtained for RCM10 and RCM20 (in this study, increases of 1.5% and 0.3%, respectively), similar to those established in other studies which show increases of 1% in RCM with 20% of CA at 90 days [22] and losses below 3% with 10 and 20% of CA at 56 days [17]. Second (intermediate content), for samples RCM30 and RCM50, less resistance was found than in the case of the UM (6% and 14%), contrary to previous studies (50% CA use shows greater fm than the reference) [16,18,22]. Finally, for the third zone (maximum content), RCM100 has a marked loss with respect to the UM (loss of 35%), coinciding with other research [4,49] (39% and 40% less than the reference at 28 days with *c*/*a* = 1:3 and 1:2). Of the three zones described, the first shows variation values which could be considered as within the natural variability of the test (see the standard deviation of the tests in Table 4); however, for the other two behavior zones the natural variability exceeds the standard deviation of the test in question, and consequently it is evident that this behavior must be attributed to the variable XX% of the RCM studied.

**Figure 6:**
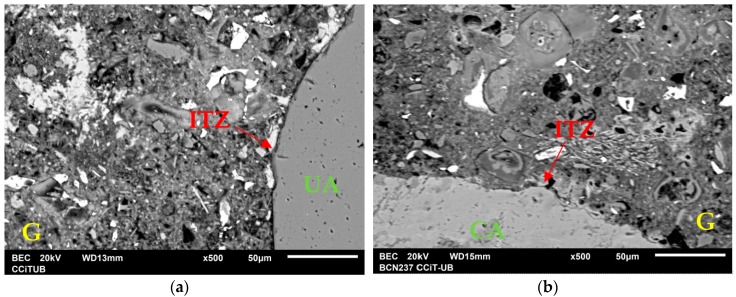
(**a**) UM (0% of CA); and (**b**) RCM10 (10% of CA).

The explanatory hypotheses of the compressive behavior for the different CA contents are: for low CA contents, there is a possibility that the strength increases are due to a pozzolanic reaction in the fine fraction of the CA, causing a “filling” effect in the matrix and a reduction of the porosity (which improves strength). The validation of this hypothesis was corroborated by observing the matrix with a JEOL JSM-6510 scanning electron microscope (SEM). In Figure 6, the UA, CA, grout (G) and the interfacial transition zone (ITZ) can be seen both in Figure 6a for UM and Figure 6b for RCM10. In Figure 6a, a wide ITZ can be seen around the UA, as well as a smoother UA profile (less adherence), while Figure 6b shows a denser and more uniform (lower porosity) matrix, a thinner ITZ and a rougher CA.

**Figure 7:**
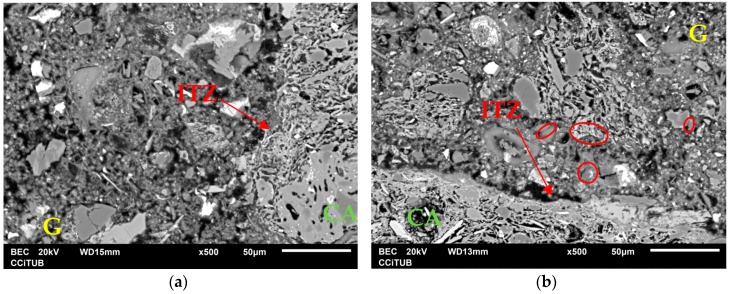
SEM of: (**a**) RCM50 (50% of CA); and (**b**) RCM100 (100% of CA).

**Figure 8:**
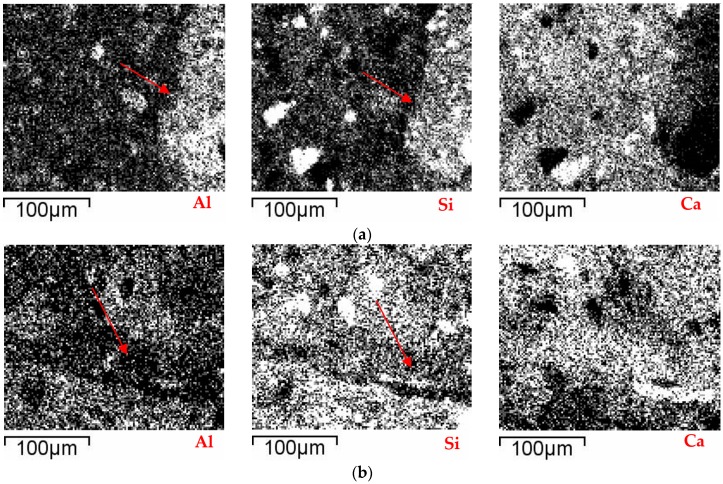
Microanalysis of components by mapping in SEM of the majority compounds: (**a**) RCM50; and (**b**) RCM100.

Additionally, another possible reason why the RCM show divergences in their strength is the method of proportioning used in their dosage (weight or volume); for example, when substituting materials according to weight a greater amount of inferior quality mortar is produced [22], due to the lower density of the recycled aggregate, which causes lower strength. Regarding the RCM with strength losses, it is suggested that the increase in the CA content causes the pozzolanic reaction of the fine fraction to be lost (or annulled), leading to the unfavorable effects of lower density and high absorption of CA becoming dominant in the resistance behavior of the matrix (weaker and more porous). In Figure 7, the samples RCM50 (Figure 7b) and RCM100 (Figure 7b) are shown; in both cases the ITZ is wider and G is disperse and non-homogeneous; in particular, in Figure 7b, a zone of high porosity can be seen around the CA, as well as the presence of microfissures (marked in the circumferences). Figure 8 shows the location of the most usual components in mortar chemistry for the two samples referred to; the images were obtained through mapping and microanalysis by X-ray diffraction (XRD) connected to the SEM used. The preferred location refers directly to the origin of the CA, and the degree and chemical composition of the hydration attained by G and its porosity; they are distinguished by the juxtaposition in the images of the increase of the ITZ (in Figure 8a,b for the compounds Al and Si, respectively) as well as an important number of microfissures (bad adherence) in the RCM with high contents (see Figure 7b).

### 3.3. Calculation of E

**Figure 9:**
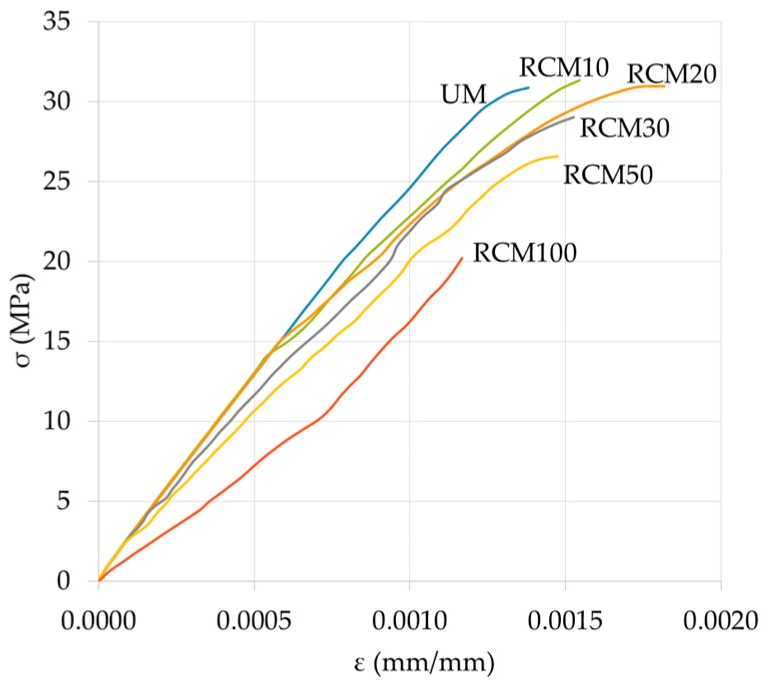
σ-ε curves of the RCM.

Figure 9 shows the curves corresponding to the RCM with the different percentages of the studied CA, up to a stress level equal to σ_max._ The *E* is determined from these curves (see test details).

In general, it can be seen that the RCM10 and RCM20 show similar values to UM (similar behavior to fm); and with regard to the rest of the mixtures (CA content ≥ 30%), the *E* shows inversely proportional losses. Specifically, for the RCM10 there is an increase of 1%, while the RCM20 has the same value of *E* (when compared to the reference); for RCM with 30%, 50% and 100%, the values of *E* suffered decreases of 8%, 17% and 46%, respectively. With the exception of RCM20, the rest of the study variables exceeded the test variability and can therefore be considered variations of the XX% of the RCM studied.

The above-mentioned trends are coherent with previous works (the case of using CA to replace cement) with different levels of loss of *E* (the maximum was 41% at 70 days) [9]. Similarly, substitutions of 0%, 10%, 20%, 30% and 40% of CA by UA with *w*/*c* mixtures of 0.4 and 0.5 show decreases of 3%, 5%, 7% and 9% at 28 days with *w*/*c* = 0.5; while for mortars with 10% and 20% of CA and *w*/*c* = 0.4 increases of 1% and 2% respectively were obtained [12], similar to this study. However, Higashiyama et al. [24] obtained values of *E* higher than the reference (2% and 5%) in mortars with 100% of CA and with 10% and 20% of CePo as a cement replacement.

Compared with studies where percentages of the UA were replaced by CA (similar to this one), the results were contradictory; RCM with 10% of CA (*c*/*a* = 1:6 and 1:4; at ages of 2–5 months) showed losses of 18% and 17%, respectively (similar values for the effect of *c*/*a*). The explanation for this was attributed to the forming of cracks, given that a lower value of *E* permits lower internal tensions for identical deformations [20]. Similarly, in another work by the same author, with replacement of 50% of CA by UA (*c*/*a* = 1:4 at ages of two and five months), the resulting losses were of 40% regarding the reference samples [18].

To sum up, it can be said that in this study the behavior of *E* is similar, in terms of its origin, its implications and its justification (the effect of the CA on the RCM), to the previously mentioned case of the property of fm (correlated and coherent properties, as occur in the UM). However, it should be pointed out that in the previous works in which the *E* of the RCM was studied [18,19], this was determined by means of resonance vibration frequency according to the French norm NF B10-511F [50]. This involved causing the specimen to vibrate until the resonance frequency was obtained, with which it was then possible to determine the dynamic elasticity modulus through an established equation. Consequently, the scarcity of studies that obtain the *E* of RCM by means of the σ-ε curve is patent. As this provides knowledge to this field of study, a cautious comparison of the various studies is required until further data are obtained. 

From the curves in Figure 9, the previously mentioned procedures were carried out in order to establish the behavior properties of σ-ε of the RCM (see Table 4, average values of each test and its standard deviation). It can be appreciated that for *T* the values obtained have two clearly defined zones, limited by RCM20: before it, increases of up to 49% are seen (the pozzolanic reaction effect of the CA); after it (high CA contents) the losses are of up to 55% (RCM100), in both cases regarding UM. Concerning *U*_r_, the observed behavior is similar to that of *T*, with the difference that after RCM20 the losses are not proportional (linear decrease of *T*); meanwhile, in the case of *U*_r_ they undergo acceleration with the increase of CA. Therefore it can be expected that in the elastic range of the RCM the effect of the increase of CA will make it more likely for them to have a normal behavior or one similar to a UM. Regarding ε_elastic_, the values obtained from the different RCM may all be considered as constants (0.0005 mm/mm), thereby establishing this as the reference value for the use, design and calculation of mortar applications. For the RCM, regarding the ε_max._, the increases obtained with respect to ε_elastic_ are on average 3.15 times bigger, with the greatest increase being in RCM20 (limit of the zone with pozzolanic reaction, with 3.92 times more). This means that the RCM would have a plastic deformation capacity of between 1.48 and 3.12 times more than, for example, the usual concrete; and comparing the average of all RCM with respect to the UM, the former will show 0.43 times less deformation. Therefore, it can be said that the RCM have a tendency to show more fragile failures than the UM. Finally, as regards the *E*_0_, there is an evident loss of rigidity for all the CA contents, which is correlational with the increase of the CA content.

**Figure 10:**
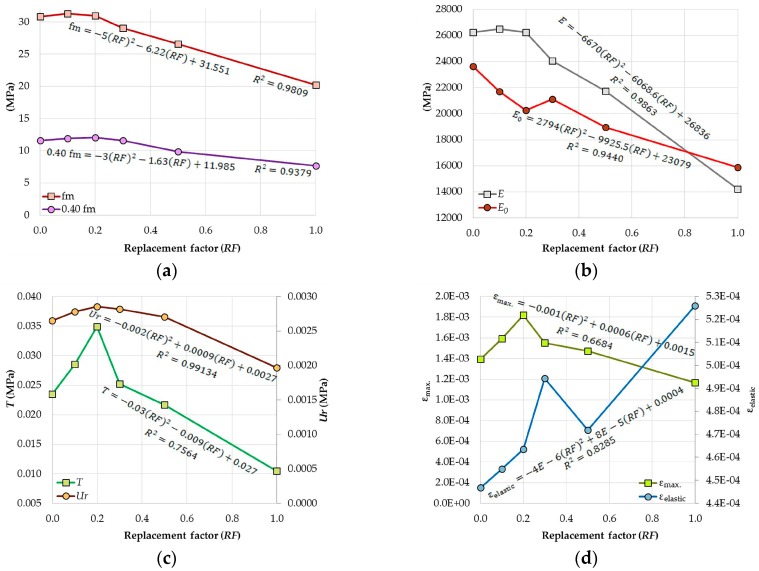
Graphs of the different properties of the RCM vs. *RF*: (**a**) *RF* vs. fm and 0.4 fm; and (**b**) *RF* vs. *E* and *E_0_*; (**c**) *RF* vs. *T* and *U_r_*; (**d**) *RF* vs. ε_max._ and ε_elastic_.

Figure 10 shows the graphs corresponding to the results of σ-ε obtained from the RCM and the equations which best adjust the trends (coefficient of determination) referring to the replacement factor (*RF*, where *RF* = content of CA/100); the use of these correlations was decided upon in order to show the significance of the *RF* with respect to the properties of the RCM. As can be seen, the graphs in Figure 10a–c and the variable ε_max._ in Figure 10d generally tend to reduce their values as the *RF* increases, their best fitting curves being represented by second degree polynomial equations. Specifically, if the equations are aimed at the particular behavior of the properties studied, they could even establish two markedly different zones; for low *RF* (≤0.20) the RCM properties show values similar to—and even greater than—those of the UM, while for *RF* ≥ 0.20 the trend could be represented by a decreasing linear equation. Finally, in the case of ε_elastic_ (Figure 10d), in contrast to all of the others, the *RF* effect generates a generally increasing equation.

### 3.4. Determining the Prediction Equations of the RCM

Once the physical, mechanical and σ-ε behavior of the different RCM had been obtained, the objective became the study and establishment of the prediction equations of behavior by means of the prescription, use and determination of the relationship of their properties, as well as the proposal of new coefficients (proper to the RCM). Similarly, numerical equations describing the curve, both in the elastic and plastic range up to the failure load, were determined for all the variables studied.

In order to obtain the prediction equations various pre-existing configurations for the usual concrete were evaluated, intending to optimize their adaptation to mortars and recycled materials; similarly (taking the previously acquired physical and mechanical properties into account) new combinations were also evaluated with the aim of finding those which best described and predicted the σ-ε behavior of the RCM. Finally, apart from establishing the refined prediction equations (*PE*_r_) (for each XX% studied in this research), these were distinguished by means of correction coefficients (*C*_C_); thus aiding their exactitude and precision in predicting the RCM.

To obtain them, it was necessary to use algorithms of numerical approximation by sequencing in cycles with correction criteria known as optimizers, in particular those provided by Solver [51]. This software forms part of the command or scenario analysis tools that aid in choosing the best method to make or determine a behavior law. Specifically, using Solver it was possible to find the theoretical constants (*T*_C_) of the conceptual models or predictive equations that represented the behavior of the real values obtained in the experimental campaign. Once the logical conditions (or limitations) of the test had been satisfied and the studio series validations had been carried out, the results obtained from the process were the coefficients that satisfied these pre-established limits. The process included:

Defining the proposed configuration to be solved (objective equation, *O*_E_). The order, position and variables to be considered were tested according to pre-established logical criteria of the UM behavior, as observed in Equation (4).

(4) $$O_{\text{E}}=\left(T_{\text{C}}\right)\;\times \;Study\;configuration$$

where *T*_C_ is conditioned to be a whole value.

The *PE*_r_ to be determined was prescribed with two coefficients or adjustment constants: the first constant (*T*_C_) was limited by the maximum and minimum possible values of the particular evaluation of each group of experimental values obtained (established on the basis of data in Table 4), and when possible this coefficient was forced to be a whole number (simplicity in the equation); the second constant, *C*_C_, corresponds to a refining of the *O*_E_, allowing it to be sensitive to XX%; see Equation (5).

(5) $$PE_{\text{r}}=\left(T_{\text{C}}\right)\times \left(C_{\text{C}}\right)\times Study\;configuration$$

Stringent conditions for analysis were limited to the following: the values of the variables considered in the *O*_E_ were limited by the maximum and minimum of each real experimental result (Table 4); the value of *O*_E_ with which the adjustment process was initiated was tested with four possible approximation processes: maximum and minimum values, average total value and the average of the maximum and minimum extremes; and all were obtained from the real experimental campaign. As an algorithm or method of solving the *O*_E_, of the algorithms available (non-linear method: for adjusting softened non-linear behavior; simple method: for adjusting linear problems; and the evolutionary method: for non-linear and disperse behavior), the non-linear method was used, as it was closest to the behavior of the real experimental values.

Finally, the following specifications were defined as parameters of the iteration of the applied algorithm: the minimum acceptable value of error in the convergence of the *O*_E_ equal to or less than 0.0001 (average figure of the last five iterations); evaluation of the iterations *i* + 1: it was established that the new value of the variables to be tested should be determined by means of a forward difference approximation; and finally the initial test values of the variables to be evaluated in the *O*_E_ were set as equal for all, with a value equal to zero (hypothesis of test values corresponding to the same study phenomenon).

When the *O*_E_ with its constant *T*_C_ has been obtained, the value of the distinctive constant *C*_C_ is determined from the relationship between the real experimental value for each sample with different XX% and the theoretical value of the established *O*_E_; giving as a result as many *O*_E_ (with distinctive *C_C_*) as RCM studied, with Equation (6).

(6) $$C_{\text{C}}=real\;experimental\;value\;of\;the\;CRMXX\%/value\;obtained\;from\;the\;O_{\text{E}}$$

where 0 ≤ *XX%* ≤ 100.

These *O*_E_ with distinctive *C*_C_ (*PE*_r_) were compared with the real experimental values, the difference being called the Error Differential (*E*_D_) of exactitude obtained from Equation (7).

(7) $$E_{\text{D}}=PE_{\text{r}}-\;real\;value\;of\;the\;experimental\;campaign\;for\;each\;CRMXX\%$$

where 0 ≤ XX% ≤ 100.

Taking into account the group of the *E*_D_ established for each *PE*_r_ and real test, their standard deviation, *S*, was determined with Equation (8) and the Relationship (*R*) of the average of *E*_D_ ($\bar{X}$) with respect to the average of the real experimental values (${\bar{X}}_{r}$) (in percentage, see Equation (9)). It is the criteria (*S* and *R*) and the agreement between the experimental and simulated group which define, firstly, the criteria for determining the best approximation process for configuring the target equation analyzed and, secondly, those for choosing, from the different configurations, the *O*_E_ which is the most appropriate, accurate and similar to the real experimental values.

(8) $$S=\sqrt{\sum_{XX\%=0}^{XX\%=100}{\left(X_{XX\%}-\bar{X}\right)}^{2}/n-1}$$

where *n* is the number of real tests carried out; $\bar{X}$ the average of the *E*_D_ and *Χ* the *E*_D_ for each XX% of the study.

(9) $$R\;\left(\%\right)=\;\left(\bar{X}/{\bar{X}}_{r}\right)\times 100$$

where $\bar{X}$ is the average of the *E*_D_ and ${\bar{X}}_{r}$ the average of the real experimental values.

From the results of the previous procedures for obtaining the *PE*_r_, the following equations were established:

The static modulus of elasticity of Equation (10), established by means of the real properties of fm and ρ, and being defined as *PE*_r_ through the use of the *C*_C_ (see Table 5, in column *E*) for each RCM. The coefficients *C*_C_ from XX% ≥ 30 show significant reductions which will establish differences in the *PE*_r_.

(10) $$E=5521C_{\text{C}}\times \sqrt{0.40\;fm\times \varrho }$$

In the specific case of the ε_elastic_, it was decided to establish two types of *PE*_r_, as the real experimental results did not converge satisfactorily for all the RCM studied; therefore, first Equation (11) is valid for 0 ≤ XX% ≤ 30, and Equation (12) is applied for 30 ≤ XX% ≤ 100. In both cases, the equations follow similar proposals of variables, and the *C*_C_ constants to be used for each RCM may be found in Table 5, column ε_elastic_.

(11) $$\varepsilon_{\mathrm{elastic}}=C_{\text{C}}\times 0.40\;fm/E$$

(12) $$\varepsilon_{\mathrm{elastic}}=0.007C_{\text{C}}\times 0.40\;fm/\sqrt{E}$$

Regarding the *U*_r_, the proposed Equation (13), along with the coefficient *C*_C_ of Table 5 column *U*_r_, allows the capacity of the different RCM to absorb or transform the energy or workload to be established. In the particular case of *C*_C_ = 100%, this is the coefficient that can cause greatest variation in this property.

(13) $$U_{r}=5C_{\text{C}}\times 0.40\;fm/E$$

For property *T*, Equation (14) has been formulated, based on a formulation of the area of a triangle, which, along with the coefficients *C*_C_, allows it to be refined for the different RCM.

(14) $$T=0.62C_{\text{C}}\times fm\times \varepsilon_{\max.}$$

**Figure 11:**
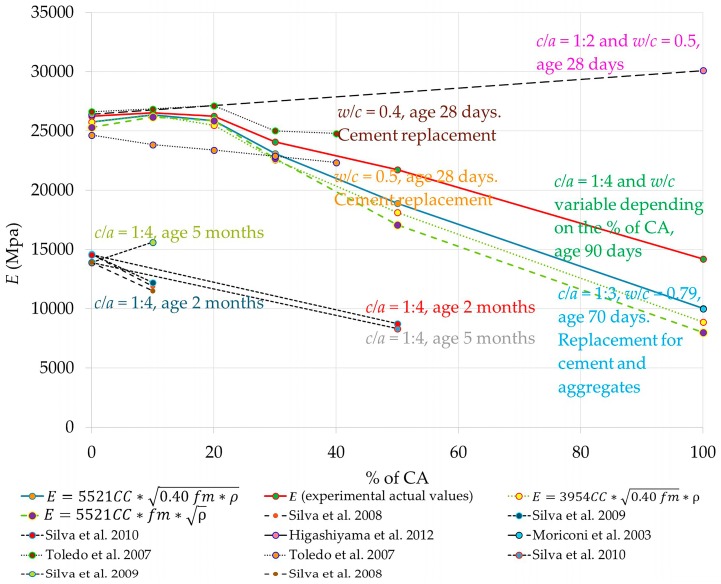
*E* prediction equation for RCM confronted with real experimental data, and reported in other investigations.

The graph in Figure 11 shows the proposed prediction equation of *E* for the RCM (10), as well as the real experimental values of the research campaign. The equation chosen was that which achieved the best convergence, and graphically it is closer to the line of the real experimental values (see the other equations considered). Similarly, the graph shows the results of other previously-mentioned studies; although, unfortunately, these were proposed with different variables and parameters (shown in the graph), which makes it difficult for the proposed equation to fit (or explain) all of them, although the trend is similar in several [18,19,20]. Further research will be necessary, and new simulations with different adjustment and refinement parameters should be performed. The established equation, therefore, could be applied to predicting *E* in the RCM with a *c*/*a* close to 1:4, for density values of the RCM in hardened state between 1.95 ≤ ρ ≤ 1.53 kg/cm^3^ and for replacement values of UA by CA between 0 ≤ XX% ≤ 100.

Table 6 shows the real experimental values of the properties studied, as well as the theoretical data calculated from the previously established simulation Equations (10)–(14). This table allows comparison of the approximations and the exactitude obtained by use of the behavior prediction equations of the RCM.

Finally, the analytical expression of the curve of the σ-ε relationship of the RCM studied was obtained with the results of Table 4 (values of *E* and *E*_0_), using in its conception the proposal by Fanella and Naaman [47] for this objective (Equation (15)).

(15) $$Y=\left[AX+BX^{2}/1+CX+DX^{2}\right]$$

In the previous equation, the constants of use (*A*, *B*, *C* and *D*, see Equations (16)–(19)) are established, between the proportions of the mechanical properties of the RCM and those of the mathematical and geometrical parameters that produce the curve of an equation fitting the behavior of σ-ε; and *X* is the value of the deformation in the graph of the real experimental *E* for the particular point to be determined on the theoretical curve.

(16) $$A_{1}=E/E_{0}$$

(17) $$B_{1}=0.45-0.41/A_{1}+0.20/{A_{1}}^{2}$$

(18) $$C_{1}=A_{1}-2$$

(19) $$D_{1}=B_{1}+1$$

Using Equation (15) to define the curve of the behavior to be studied and the real experimental values of the RCM, Equation (20) (*O*_E_) is defined, which has the constant belonging to the RCM (*T*_C_) family of data (see Table 7); its determination was obtained through a similar calculation process with the Solver tool.

(20) $$O_{\text{E}}=\left(T_{\text{C}}\right)\times \left[AX+BX^{2}/1+CX+DX^{2}\right]$$

where *X* is equal to ε.

To achieve better prediction of the different RCM, a *PE*_r_ (Equation (21)) has been established by means of the particular constants *C*_C_ (Table 8) of each XX%, to which the previously indicated process was applied.

(21) $$PE_{\text{r}}=\left(T_{\text{C}}\right)\times \left(C_{\text{C}}\right)\times \left[AX+BX^{2}/1+CX+DX^{2}\right]$$

Once the processes had been carried out, it was necessary to establish three different equations (and *T*_C_) to better adjust the numerical predictions to the real experimental results: two equations were established with respect to the prediction of σ in the σ-ε curve of the RCM until the elastic range (σ_elastic_); one for 0 ≤ XX% ≤ 50 and another for XX% = 100. For σ of the curve to maximum of failure (σ_max._), a single equation was established for all of the XX% (of less precision) (see Table 7 and Table 8).

**Figure 12:**
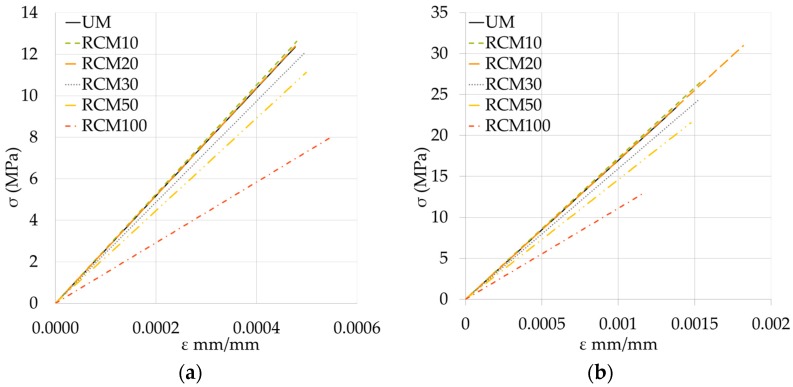
Curve σ-ε simulated for the RCM: (**a**) up to σ_elastic_; and (**b**) up to σ_max._

The σ-ε curves of the RCM studied by means of the previously-determined simulation equations are shown in Figure 12. In both cases, their profiles maintain the order established according to XX% used; likewise, establishing a grouping between the curves showing similar behavior (0 ≤ XX% ≤ 20), afterwards distancing them proportionally in accordance with XX% (30 ≤ XX% ≤ 100).

## 4. Conclusions

Based on the experimental data obtained and the numerical processes carried out, the following conclusions can be presented:

The density of the RCM is less than that of the UM, this being inversely correlative to the content of CA; this reduction in density has been attributed to the low density of the CA.

The behavior of the mechanical property of fm responds to three different groupings in terms of CA replacement: with small replacements (10% to 20%), the RCM show similar behavior, even managing to occasionally exceed the UM; for intermediate content replacements (30% to 50%), losses in strength of between 6% and 14% may occur; and, finally, for a total replacement, the losses can be significant with respect to the UM (35% less). In the previous behavior, the study of the ITZ in SEM images validates the hypothesis that the strength increases of the RCM can be attributed to the possible pozzolanic reaction of the CA, causing a “filling” effect in the matrix and reducing its porosity; on the contrary, a less dense ITZ can be seen for high contents, possibly meaning greater porosity in this area, which, combined with the low density of the aggregates, leads to a lower fm in RCM with high contents. Similarly, it has been shown that the mineralogical composition (established by means of XRD) of the CA is not involved in the possible later variations in the RCM behavior.

The σ-ε behavior of RCM is similar to that shown by UM, and is the closest to the RCM10 and RCM20; in the case of σ, the RCM10 obtained values even higher than the UM. As for ε, this increases as the percentage of CA is increased, up to a maximum of 20%; with CA contents CA ≥ 30%, losses inversely proportional to the content of the CA are obtained. The results of *E*, obtained from the σ-ε curves, show similar behavior to that shown by the property of fm; for the RCM10 an increase of 1%, for the RCM20 values similar to the UM, and for RCM with CA ≥ 30% the losses are established in the range of 8% to 46%. The previous behavior has been validated as part of the variables studied, and not as the result of its statistical variability, thus lending strength to the research.

The results of the properties of the RCM that were obtained by means of the σ-ε curves have shown increases, while the CA content has been increased until 20%; when the replacement passes 30% the values of each of the properties is inferior regarding the UM. This data has made it possible to formulate predictive equations regarding the percentage of CA, which (quickly and simply) provide values close to the different RCM.

By means of numerical analysis, two constants for use in predictive equations have been obtained, which allow the different properties of the RCM to be related; the first of them is the theoretic constant which represents the behavior of the real values obtained during the experimental campaign of the CA, and the other allows exactitude and precision for each of the RCM with its respective percentage of CA. Specifically, for the predictive equation of *E*, this cannot be corroborated by using the results of previous studies, as these have been proposed with different variables and parameters, although in some cases similar trends are evident.

The analytical expressions obtained from the σ-ε curve of the studied RCM allow the curves in the elastic range to be predicted, up to their failure point; thereby easing the study, design and calculation of the constructive elements that include these mortars.

## Figures and Tables

**Table 1:** Physical properties of aggregates.

Property ^1^	CA	UA
Without adjusted granulometric profiles		
*M*_OD_ (kg/m^3^)	1182.0	1735.1
*M*_SSD_ (kg/m^3^)	1399.1	1860.8
Void content (%)	35.3	32.9
With adjusted granulometric profiles		
*D*_OD_ (kg/m^3^)	1820.9	2581.6
*D*_SSD_ (kg/m^3^)	2155.4	2623.6
Water absorption coefficient (%)	18.4	1.6
Fineness modulus materials	2.8	2.4
Particles < 75 µm (sieve No. 200) (%)	8.2	2.9

^1^ According to ASTM (C128 [34], C136 [35] and C117 [36]).

**Table 2:** Characteristic and proportions of the mixtures.

Materials (g)		Classification and Proportions of the Mixtures					
		UM	RCM10	RCM20	RCM30	RCM50	RCM100
Water		334	390	355	373	397	476
Cement		400	433	381	372	348	323
UA ^1^	<sieve No. 30	800	780	610	521	348	0
	>sieve No. 30	800	780	610	521	348	0
CA ^1^	<sieve No. 30	0	69	122	179	278	517
	>sieve No. 30	0	104	183	268	417	775

^1^ Dry condition.

**Table 3:** Density in hardened state of the RCM.

Study Variables	ρ (g/cm^3^)
UM	1.950
RCM10	1.948
RCM20	1.894
RCM30	1.864
RCM50	1.798
RCM100	1.529

**Table 4:** Properties of σ-ε of the RCM.

Study Variables	fm (MPa)	0.40 fm (MPa)	*E* (MPa)	*T* (MPa)	*U*_r_ (MPa)	ε_elastic_ (mm/mm)	ε_max._ (mm/mm)	*E*_0_ (MPa)
UATM	28.77 ± 2.28	11.51 ± 0.91	26252 ± 41	0.024 ± 1.8 × 10^−4^	0.003 ± 2.53 × 10^−4^	0.0004 ± 2.18 × 10^−5^	0.0014 ± 2.03 × 10^−5^	23619 ± 1004
CRM10	30.58 ± 1.12	12.23 ± 0.45	26514 ± 53	0.029 ± 1.77 × 10^−3^	0.003 ± 7.29 × 10^−5^	0.0005 ± 6.46 × 10^−6^	0.0016 ± 6.53 × 10^−5^	21710 ± 520
CRM20	30.95 ± 1.44	12.38 ± 0.58	26251 ± 31	0.035 ± 7 × 10^−4^	0.003 ± 1.4 × 10^−4^	0.0005 ± 2 × 10^−5^	0.0018 ± 3.1 × 10^−5^	20249 ± 542
CRM30	30.10 ± 0.99	12.04 ± 0.40	24064 ± 6	0.025 ± 1.97 × 10^−4^	0.003 ± 8.92 × 10^−5^	0.0005 ± 1.45 × 10^−5^	0.0016 ± 3.46 × 10^−5^	21107 ± 304
CRM50	26.76 ± 2.48	10.71 ± 0.99	21731 ± 25	0.022 ± 5 × 10^−4^	0.003 ± 1 × 10^−4^	0.0005 ± 2.98 × 10^−5^	0.0015 ± 2.06 × 10^−6^	18940 ± 745
CRM100	20.56 ± 0.34	8.23 ± 0.14	14194 ± 30	0.010 ± 8 × 10^−4^	0.002 ± 1.5 × 10^−4^	0.0005 ± 3 × 10^−5^	0.0012 ± 2 × 10^−5^	15891 ± 643

**Table 5:** *C*_C_ values for application in *PE*_r_ equations of the RCM.

Study Variables	Corrector Coefficient (*C*_C_ for Mechanical Properties of RCM)			
	*E*	ε_elastic_	*U*_r_	*T*
UM	0.9817	0.9380	1.0086	0.6651
RCM10	0.9915	0.9553	1.0572	0.8075
RCM20	0.9817	0.9734	1.0855	0.9880
RCM30	0.8999	1.0380	1.0716	0.7127
RCM50	0.8126	0.9910	1.0291	0.6129
RCM100	0.5308	1.1043	0.7480	0.2968

**Table 6:** Values of the experimental properties and those determined through simulation.

Study Variables	*E* (MPa)		ε_elastic_ (mm/mm)		*U*_r_ (MPa)		*T* (MPa)	
	Expt.	Simul.	Expt.	Simul.	Expt.	Simul.	Expt.	Simul.
UM	26,252	25,771	0.0004	0.0004	0.003	0.002	0.024	0.018
RCM10	26,515	26,334	0.0005	0.0004	0.003	0.002	0.029	0.025
RCM20	26,251	25,493	0.0005	0.0004	0.003	0.002	0.035	0.035
RCM30	24,065	22,578	0.0005	0.0005	0.003	0.003	0.025	0.020
RCM50	21,731	18,127	0.0005	0.0005	0.003	0.002	0.022	0.015
RCM100	14,194	8883	0.0005	0.0005	0.002	0.002	0.010	0.004

**Table 7:** Values of the constants for each of the curves.

*PE*_r_ of the Curve σ-ε	Application	*T*_C_ (for the σ-ε Curve of the RCM)	*A*	*B*	*C*	*D*
*PE*_r_ for σ_elastic_	0 ≤ XX% ≤ 50	16,337	0.893	0.242	−0.704	1.253
*PE*_r_ for σ_elastic_	XX% = 100%	16,337	1.245	0.253	−0.704	1.242
*PE*_r_ for σ_max._	0 ≤ XX% ≤ 100	13,113	1.179	0.253	−0.704	1.242

**Table 8:** Values C_C_ for the different percentages of CA.

XX%	Corrector Coefficient (*C*_C_ for σ-ε Curve of the RCM)	
	*C*_C_ for *PE*_r_ up to σ_elastic_	*C*_C_ for *PE*_r_ up to σ_max._
0	1.7739	1.0962
10	1.8005	1.1126
20	1.7788	1.0992
30	1.6677	1.0306
50	1.5276	0.9440
100	0.7174	0.7174

## Abbreviations

The following abbreviations are used in this manuscript:

σ-ε: Stress-strain
CA: Recycled aggregates of ceramic
UA: Usual aggregates
RCM: Recycled ceramic mortars
CePo: Ceramic powder
*c*/*a*: Cement/aggregate
*w*/*c*: Water/cement
fm: Compressive strength
PS: Particle size
*E*: Module of elasticity or Young
ε_elastic_: Elastic deformation
0.40 fm: 40% of the maximum load of failure
ε_max._: Maximum deformation of failure
*U*_r_: Resiliency
*T*: Toughness
*M*_OD_: Bulk density in oven-dry condition
*M*_SSD_: Bulk density in saturated-surface-dry condition
*D*_OD_: Density in oven-dry condition
*D*_SSD_: Density in saturated-surface-dry condition
UM: Usual mortar
SEM: scanning electron microscope
G: Grout
ITZ: Interfacial transition zone
XRD: X-ray diffraction
*RF*: Replacement factor
*PE*_r_: Refined prediction equations
*C*_C_: Correctors coefficients
*T*_C_: Theoretical constants
*O*_E_: Objective equation
*E*_D_: Error Differential
*S*: Standard deviation
σ_elastic_: Stress in the elastic range
σ_max._: Up to maximum stress of failure
